# Supplementary material for: Mechanisms governing the pioneering and redistribution capabilities of the non-classical pioneer PU.1
Source: Nat Commun. 2020 Jan 21;11:402. doi: 10.1038/s41467-019-13960-2 (PMC6972792; doi:10.1038/s41467-019-13960-2)
Supplement: Supplementary file 7 — Source data [file 41467_2019_13960_MOESM7_ESM.zip › Source_Data/Figure6/Figure6B_MotifScanOutput/FLI1_PU.1/homerResults/motif3.similar.html]

motif3

## Information for motif3

C
G
T
A
A
T
C
G
G
C
A
T
C
T
G
A
T
C
G
A
A
G
T
C
A
G
T
C
C
T
G
A
A
G
T
C
G
C
T
A
  
Reverse Opposite:  

C
G
A
T
A
C
T
G
A
G
C
T
A
C
T
G
A
C
T
G
A
G
C
T
G
A
C
T
C
G
T
A
T
A
G
C
G
C
A
T
  

|  |  |
| --- | --- |
| p-value: | 1e-592 |
| log p-value: | -1.365e+03 |
| Information Content per bp: | 1.654 |
| Number of Target Sequences with motif | 3639.0 |
| Percentage of Target Sequences with motif | 36.58% |
| Number of Background Sequences with motif | 4577.0 |
| Percentage of Background Sequences with motif | 12.54% |
| Average Position of motif in Targets | 102.1 +/- 49.6bp |
| Average Position of motif in Background | 99.2 +/- 63.5bp |
| Strand Bias (log2 ratio + to - strand density) | 0.0 |
| Multiplicity (# of sites on avg that occur together) | 1.23 |
| Motif File: | file (matrix) reverse opposite |

### Similar de novo motifs found

|  |  |  |  |  |  |  |  |
| --- | --- | --- | --- | --- | --- | --- | --- |
| Rank | Match Score | Redundant Motif | P-value | log P-value | % of Targets | % of Background | Motif file |
| 1 | 0.991 | C G A T C T A G G A C T T A C G C T A G A G C T G A C T C G A T G C T A | 1e-568 | -1308.990689 | 35.31% | 12.08% | motif file (matrix) |
| 2 | 0.983 | C G A T C T A G A G C T A C T G A C T G A G C T A G C T C G T A | 1e-567 | -1306.998789 | 33.90% | 11.18% | motif file (matrix) |
| 3 | 0.984 | T G A C T A C G T A G C G C T A T C G A T C G A A G T C A G T C C T G A T G A C G C T A | 1e-545 | -1255.698407 | 34.50% | 11.91% | motif file (matrix) |
| 4 | 0.961 | C A T G C A T G C A T G A C T G G A C T C G A T A T C G G C A T C A T G A C T G A C G T G A C T | 1e-459 | -1056.933030 | 30.59% | 10.74% | motif file (matrix) |
| 5 | 0.960 | T G C A C T A G T C A G G T C A T C G A T C G A A G T C A G T C G C T A T A G C G C T A C T A G T G A C | 1e-441 | -1015.576911 | 22.44% | 6.07% | motif file (matrix) |
| 6 | 0.969 | A C G T A C T G A G C T A C T G A C T G A C G T G A C T | 1e-432 | -996.223320 | 27.04% | 8.90% | motif file (matrix) |
| 7 | 0.897 | G A C T C A G T A C T G A G T C A T C G A C T G G A C T C G A T C G T A | 1e-394 | -908.359118 | 26.98% | 9.48% | motif file (matrix) |
| 8 | 0.797 | A T C G G T C A G C A T C T A G A C G T C A T G A T C G A G C T | 1e-349 | -805.013097 | 25.40% | 9.23% | motif file (matrix) |
| 9 | 0.935 | C A T G A G C T C T A G A C T G A G C T C G A T C G T A | 1e-337 | -778.007569 | 47.79% | 26.67% | motif file (matrix) |
| 10 | 0.814 | C G T A A G T C A G T C C T A G A T G C G T C A A C T G | 1e-267 | -614.809323 | 25.46% | 10.89% | motif file (matrix) |
| 11 | 0.838 | G A C T C A G T C T A G G A T C C A T G A C T G A G C T C G A T | 1e-246 | -568.699952 | 55.74% | 36.92% | motif file (matrix) |
| 12 | 0.872 | T G C A C T G A A T C G G C T A T G C A T C G A A T G C A G T C C T G A G T A C G C T A T G A C | 1e-235 | -542.908726 | 19.12% | 7.26% | motif file (matrix) |
| 13 | 0.895 | G A C T A G T C G A C T A G C T C A G T T A C G G T C A C G A T A C T G C G A T A C T G C T A G A G C T A G C T | 1e-194 | -448.640828 | 11.08% | 3.13% | motif file (matrix) |
| 14 | 0.776 | C A T G A T G C A G T C A G T C C G T A A G T C C G T A | 1e-119 | -274.068445 | 31.72% | 20.38% | motif file (matrix) |
| 15 | 0.754 | A C G T C G A T A G C T G C T A C G A T C T A G G C A T C T A G A C T G A G T C | 1e-114 | -264.768746 | 6.67% | 1.89% | motif file (matrix) |
| 16 | 0.854 | C G A T C A T G G C A T C G A T A T C G A C G T T A C G T C A G A C G T T C G A C T G A | 1e-93 | -214.233866 | 7.64% | 2.80% | motif file (matrix) |
| 17 | 0.767 | G C A T G A C T C T A G A C G T T A C G C T A G A G T C T C A G T G C A | 1e-89 | -206.339488 | 18.19% | 10.44% | motif file (matrix) |
| 18 | 0.757 | T G C A C T G A C T G A G T A C G T C A T G C A C G T A C T G A G T A C G A T C G C T A A T G C C G T A C T A G | 1e-19 | -44.899460 | 1.60% | 0.59% | motif file (matrix) |
